# Development, Identification, and Application of a Germplasm Specific SCAR Marker for *Dendrobium officinale* Kimura et Migo

**DOI:** 10.3389/fpls.2021.669458

**Published:** 2021-05-14

**Authors:** Kaixin Zheng, Yuchen Cai, Weijie Chen, Yadi Gao, Jingjing Jin, Huizhong Wang, Shangguo Feng, Jiangjie Lu

**Affiliations:** ^1^College of Life and Environmental Sciences, Hangzhou Normal University, Hangzhou, China; ^2^Zhejiang Provincial Key Laboratory for Genetic Improvement and Quality Control of Medicinal Plants, Hangzhou Normal University, Hangzhou, China; ^3^College of Bioscience and Biotechnology, Hunan Agricultural University, Changsha, China

**Keywords:** *Dendrobium officinale*, DNA markers, SCoT, SCAR marker, species identification

## Abstract

The stems of *Dendrobium officinale* have been used as a rare and valuable Chinese tonic medicine, known as “Tiepi Fengdou”, since the Qing dynasty. Because of the increased market demand and continued exploitation of this plant, the reserves of wild *D. officinale* resources have been depleted, and *D. officinale* products on the market are being increasingly adulterated. Such changes have strongly affected the sustainable utilization of this valuable medicinal plant resource and the development of related industries. In this study, a species-specific DNA marker was developed for the rapid and accurate authentication of *D. officinale*. In total, 36 start codon-targeted (SCoT) polymorphism primers were screened in 36 definite *Dendrobium* species, and a distinct species-specific DNA amplicon (SCoT13-215) for *D. officinale* was obtained. After the sequence was cloned and sequenced, a sequence-characterized amplified region marker was developed (named SHF/SHR) and validated through PCR amplification of all 38 *Dendrobium* samples. The marker’s specificity for *D. officinale* was confirmed through the consistent amplification of a clear 197-bp band. This SCAR marker can be used to rapidly, effectively, and reliably identify *D. officinale* among various *Dendrobium* species and may play an important role in ensuring the quality of medicinal preparations and protecting the germplasm of this important medicinal species.

## Introduction

*Dendrobium officinale* Kimura et Migo is an important orchid plant endemic to China that has been identified separately from other *Dendrobium* species in the Pharmacopeia of the People’s Republic of China (2010 Edition). This plant is primarily distributed in Yunnan, Guangxi, Zhejiang, Anhui, Fujian, and Sichuan Provinces of China (Tsi et al., 1999; Chinese Pharmacopoeia Commission, 2015; Teixeira da Silva et al., 2016). The stems of *D. officinale* form a rare and valuable Chinese herb that is highly valued for its promising medicinal functions (Ding et al., 2003; Feng et al., 2017; Lu et al., 2018). The *D. officinale*-derived herb is known as “Tiepi Fengdou”, and the international medical plant community refers to this herb as a “medicinal giant panda”. *D. officinale* contains abundant polysaccharides, dendrobium alkaloids, flavonoids, and other bioactive substances, which are beneficial to human stomach health, exhibiting such activities as clearing heat and toxic material, enhancing immunity, reducing blood sugar concentration, and delaying aging, and this plant is recognized as a high-end health care product (Wei et al., 2016; Tang et al., 2017; Li et al., 2019). *D. officinale* has high medicinal value and is a valuable plant resource for this traditional Chinese medicine. Therefore, this plant has high research value and broad development and utilization prospects.

However, due to the increasing market demand, the wild germplasm resources of *D. officinale* have been over-harvested which, together with the destruction of the natural habitat, has caused wild *D. officinale* resources to be critically endangered (Teixeira da Silva et al., 2016). The scarcity of *D. officinale* has resulted in medicinal *D. officinale* materials being mixed and adulterated with other materials, particularly other *Dendrobium* species, in clinical practice (Feng et al., 2015b; Zhu et al., 2018). Because of the similarities in morphological characteristics among *Dendrobium* species, identification is difficult and their use is confusing, with “synonym” and “homonym” phenomena being commonly observed (Jin and Huang, 2015). More seriously, some illegal elements have been offered as legitimate resources, which leads to low-quality material. These problems affect the protection of *D. officinale* resources and the development of the *D. officinale* industry (Tang et al., 2017; Cheng et al., 2019). Thus, to better protect and clinically utilize *D. officinale* resources, it is very important to identify a rapid and accurate method to identify *D. officinale* from its adulterants.

Initially, morphological (Wahba et al., 2014; Moudi and Go, 2017), microscopic (Zhao et al., 2017, 2018), or chemical analyses (Luo et al., 2013; Ye et al., 2017) were utilized for herb identification; however, in the majority of cases, these analyses are inadequate to correctly identify plant species (Teixeira da Silva et al., 2016; Kumar et al., 2018). Moreover, these morphological, microscopic, and chemical indicators are easily affected by environmental factors. Compared with the above-mentioned traditional plant authentication methods, DNA molecular markers can be used to detect organisms, tissues, organs, and even cells at different developmental stages (Feng et al., 2015a; Teixeira da Silva et al., 2016). The number of DNA molecular markers covering the whole genome is large, and they present high polymorphic and genetic stability; their use is not limited by environmental factors or gene expression levels. These markers have been widely used in molecular identification, phylogenetic evolution, and genetic diversity analyses of plant species (Teixeira da Silva et al., 2016; Lu et al., 2018). To date, numerous DNA marker techniques, including random amplified polymorphic DNA (RAPD) (Ding et al., 2009; Khosravi et al., 2009; Xue et al., 2010), amplified fragment length polymorphism (AFLP) (Li et al., 2008; Wahba et al., 2014), inter simple sequence repeat (ISSR) (Shen et al., 2006; Wang et al., 2009), simple sequence repeat (SSR) (Lu et al., 2012, 2013) and sequence-related amplified polymorphism (SRAP) (Ding et al., 2008), have been utilized for genetic correlation, mapping and diversity studies of *Dendrobium* species. Sequence-characterized amplified regions (SCARs) are monologs codominant markers that are screened using PCRs with a pair of specific oligonucleotide primers designed from a specific nucleotide sequence generated by RAPD, AFLP, ISSR and inter-retrotransposon amplified polymorphism (IRAP) techniques (Marieschi et al., 2016; Kumar et al., 2018). Compared with conventional molecular markers, such as RAPDs, AFLPs, ISSRs, and SSRs, SCAR markers are less sensitive to reaction conditions and are more reproducible because of their high levels of specificity. The use of SCAR markers is highly convenient and provides accurate fingerprint identification at the inter- and/or intraspecific level (Marieschi et al., 2016; Kumar et al., 2018; Yang et al., 2019). The natural distribution of *D. officinale* is often complex (Jin and Huang, 2015), the SCAR markers can play a primary role in distinguishing with other *Dendrobium* germplasm, which will continue help conservation an utilization of *D. officinale*.

Start codon-targeted (SCoT) markers, developed based on the translation start codon, are PCR-based gene-targeted markers (Collard and Mackill, 2009). SCoT markers use a single primer that amplifies the genomic region based on the conserved region surrounding the translation initiation codon ATG (Mulpuri et al., 2013; Feng et al., 2015a). SCoT markers can be used as powerful tools in the characterization of germplasm collections; their applications may include screening for genetic diversity (Igwe et al., 2017), identifying species (Hao et al., 2018), and performing phylogenetic studies (Jalilian et al., 2018). In this study, we report the suitability of polymorphic SCoT markers in developing a species-specific SCAR marker. Species-specific SCAR markers have been applied to identify many different important medicinal herbs from their close relatives or adulterants (Devaiah and Venkatasubramanian, 2008; Jiang et al., 2018). Different medicinal herbs act in different medicinal pathways; therefore, it is highly important to establish a stable and effective identification method for quality control, which necessitates developing a rapid and reliable method for the identification of *D. officinale*.

## Materials and Methods

### Plant Material and DNA Isolation

A total of 36 definite *Dendrobium* species collected from their primary distribution areas in China were used to screen for specific markers by mixing fresh leaves from 3 to 4 individuals (Table 1). Furthermore, 15 *D. officinale* populations were selected for validating the SCAR marker by using fresh leaves from at least 10 individuals (Table 2). These species were identified and authenticated by HW after flowering. All voucher specimens of the collected accessions were deposited at the Zhejiang Provincial Key Laboratory for Genetic Improvement and Quality Control of Medicinal Plants, Hangzhou Normal University. The experimental research on *Dendrobium* species was in keeping with the guidelines of Hangzhou Normal University. Total genomic DNA was extracted from the fresh leaves of the collected samples as described in a previous study (Feng et al., 2013). The integrity and quality of DNA were determined using 0.8% agarose gel electrophoresis, and the genomic DNA concentration was measured using a UV spectrometer. Stock DNA was diluted to a working solution of 50 ng/μL.

**TABLE 1:** The information for all *Dendrobium* samples used in the SCoT study.

Sample number	Species name	Origin	Voucher specimen ID*
1	*Dendrobium officinale* Kimura et Migo	Yunnan, China	HZHERB 186
2	*Dendrobium officinale* Kimura et Migo	Guangxi, China	HZHERB 173
3	*Dendrobium officinale* Kimura et Migo	Zhejiang, China	HZHERB 190
4	*Dendrobium nobile* Lindl.	Yunnan, China	HZHERB 044
5	*Dendrobium loddigesii* Rolfe	Yunnan, China	HZHERB 040
6	*Dendrobium fimbriatum* Hook.	Guangxi, China	HZHERB 026
7	*Dendrobium chrysanthum* Wall. ex Lindl.	Guangxi, China	HZHERB 020
8	*Dendrobium hancockii* Rolfe	Yunnan, China	HZHERB 032
9	*Dendrobium harveyanum* Rchb. f.	Yunnan, China	HZHERB 033
10	*Dendrobium brymerianum* Rchb. f.	Yunnan, China	HZHERB 018
11	*Dendrobium gibsonii* Lindl.	Yunnan, China	HZHERB 029
12	*Dendrobium capillipes* Rchb.	Guangdong, China	HZHERB 019
13	*Dendrobium dixanthum* Rchb.	Yunnan, China	HZHERB 024
14	*Dendrobium heterocarpum* Lindl.	Yunnan, China	HZHERB 035
15	*Dendrobium falconeri* Hook.	Guangdong, China	HZHERB 025
16	*Dendrobium wardianum* Warner	Yunnan, China	HZHERB 050
17	*Dendrobium pendulum* Roxb.	Yunnan, China	HZHERB 047
18	*Dendrobium devonianum* Paxt.	Yunnan, China	HZHERB 023
19	*Dendrobium aphyllum* (Rohb.) C. E. Fishcher	Guangxi, China	HZHERB 016
20	*Dendrobium primulinum* Lindl.	Yunnan, China	HZHERB 048
21	*Dendrobium crystallinum* Tchb. F.	Guangxi, China	HZHERB 022
22	*Dendrobium crepidatum* Lindl. ex Paxt.	Yunnan, China	HZHERB 021
23	*Dendrobium moniliforme* (Linn.) Sw.	Yunnan, China	HZHERB 042
24	*Dendrobium guangxiense* S. J. Cheng et C. Z. Tang	Yunnan, China	HZHERB 031
25	*Dendrobium densiflorum* Lindl.	Guangdong, China	HZHERB 006
26	*Dendrobium lindleyi* Steudel	Guangdong, China	HZHERB 007
27	*Dendrobium chrysotoxum* Lindl.	Yunnan, China	HZHERB 008
28	*Dendrobium thyrsiflorum* Rchb. f. ex André	Yunnan, China	HZHERB 009
29	*Dendrobium hercoglossum* Rehb. f.	Guizhou, China	HZHERB 004
30	*Dendrobium aduncum* Wall. ex Lindl.	Guangdong, China	HZHERB 005
31	*Dendrobium stuposum* Lindl.	Yunnan, China	HZHERB 075
32	*Dendrobium goldschmidtianum* Kraenzlin, Repert.	Guangdong, China	HZHERB 076
33	*Dendrobium longicornu* Lindl.	Guangxi, China	HZHERB 059
34	*Dendrobium williamsonii* Day et Rchb. F.	Guangxi, China	HZHERB 060
35	*Dendrobium strongylanthum* Rchb. F.	Yunnan, China	HZHERB 068
36	*Dendrobium hainanense* Rolfe	Hainan, China	HZHERB 073
37	*Dendrobium christyanum* Rchb. F.	Yunnan, China	HZHERB 053
38	*Dendrobium denneanum* Kerr.	Guangdong, China	HZHERB 017

*All voucher specimens were deposited at the Zhejiang Provincial Key Laboratory for Genetic Improvement and Quality Control of Medicinal Plants, Hangzhou Normal University.

**TABLE 2:** List of *Dendrobium officinale* populations used for SCAR markers validation.

Population number	Original collection site	Voucher specimen ID*
1	Wenshan, Yunnan, China	HZHERB 186
2	Wenshan, Yunnan, China	HZHERB 127
3	Simao, Yunnan, China	HZHERB 131
4	Simao, Yunnan, China	HZHERB 168
5	Rongxian, Guangxi, China	HZHERB 173
6	Rongxian, Guangxi, China	HZHERB 111
7	Jinhua, Zhejiang, China	HZHERB 102
8	Jinhua, Zhejiang, China	HZHERB 108
9	Hangzhou, Zhejiang, China	HZHERB 190
10	Hangzhou, Zhejiang, China	HZHERB 175
11	Hangzhou, Zhejiang, China	HZHERB 176
12	Wenzhou, Zhejiang, China	HZHERB 172
13	Wenzhou, Zhejiang, China	HZHERB 178
14	Tiantai, Zhejiang, China	HZHERB 166
15	Xianju, Zhejiang, China	HZHERB 167

*All voucher specimens were deposited at the Zhejiang Provincial Key Laboratory for Genetic Improvement and Quality Control of Medicinal Plants, Hangzhou Normal University.

### PCR Amplification With SCoT Primers

For the initial screening, a total of 36 SCoT primers (Table 3) were synthesized by Shanghai Sangon Biological Engineering Technology and Service Co., Ltd., Shanghai, China, based on the study by Collard and Mackill (2009). PCR analysis was performed in a total volume of 20 μL containing 2 μL 1 × PCR buffer [100 mM (NH_4_)_2_SO_4_, 100 mM KCl, 1% Triton X-100, pH 8.8], 2 μL Mg^2+^ (25 mM), 0.8 μL dNTPs (10 mM), 1 μL primer (10 μM), 0.5 μL *Taq* DNA polymerase (2 U/μL) (TaKaRa Bio., Kyoto, Japan) and 50 ng genomic DNA template. Amplification reactions were performed in a thermal cycler (MJ Research PTC-100, Waltham, MA, United States) using the following parameters: 5 min at 94°C; 35 cycles of 1 min at 94°C, 1 min at 50–60°C (depending on the annealing temperature of each primer) and 2 min at 72°C; and a final extension at 72°C for 10 min. After purification with an EZ-10 spin column PCR product purification kit (Sangon Biotech, Shanghai, China), the PCR products were electrophoresed on a 1.5% agarose gel alongside *Trans2K* DNA markers (TransGen Biotech Co., Ltd., Beijing, China), followed by staining with GelStain (TransGen Biotech Co., Ltd.) and photography was performed using a Molecular Imager^®^ Gel Doc^TM^ XR + System with Image Lab^TM^ Software (Bio-Rad, Philadelphia, PA, United States).

**TABLE 3:** 36 SCoT primer sequences used in this study.

Primer name	Primer sequence (5′–3′)	Tm (°C)	GC content (%)
SCoT1	CAACAATGGCTACCACCA	49.86	50
SCoT2	CAACAATGGCTACCACCC	50.73	56
SCoT3	CAACAATGGCTACCACCG	51.27	56
SCoT4	CAACAATGGCTACCACCT	49.5	50
SCoT5	CAACAATGGCTACCACGA	50.1	50
SCoT6	CAACAATGGCTACCACGC	52.05	56
SCoT7	CAACAATGGCTACCACGG	51.27	56
SCoT8	CAACAATGGCTACCACGT	50.41	50
SCoT9	CAACAATGGCTACCAGCA	50.32	50
SCoT10	CAACAATGGCTACCAGCC	51.19	56
SCoT11	AAGCAATGGCTACCACCA	51.37	50
SCoT12	ACGACATGGCGACCAACG	55.93	61
SCoT13	ACGACATGGCGACCATCG	55.39	61
SCoT14	ACGACATGGCGACCACGC	58.58	67
SCoT15	ACGACATGGCGACCGCGA	59.85	67
SCoT16	ACCATGGCTACCACCGAC	54.05	56
SCoT17	ACCATGGCTACCACCGAG	53.71	61
SCoT18	ACCATGGCTACCACCGCC	57.09	67
SCoT19	ACCATGGCTACCACCGGC	57.09	67
SCoT20	ACCATGGCTACCACCGCG	57.53	67
SCoT21	ACGACATGGCGACCCACA	56.65	61
SCoT22	AACCATGGCTACCACCAC	51.85	56
SCoT23	CACCATGGCTACCACCAG	52.43	61
SCoT24	CACCATGGCTACCACCAT	51.58	56
SCoT25	ACCATGGCTACCACCGGG	56.35	67
SCoT26	ACCATGGCTACCACCGTC	54.05	61
SCoT27	ACCATGGCTACCACCGTG	54.37	61
SCoT28	CCATGGCTACCACCGCCA	57.1	67
SCoT29	CCATGGCTACCACCGGCC	57.9	72
SCoT30	CCATGGCTACCACCGGCG	58.32	72
SCoT31	CCATGGCTACCACCGCCT	56.77	67
SCoT32	CCATGGCTACCACCGCAC	55.94	67
SCoT33	CCATGGCTACCACCGCAG	55.62	67
SCoT34	ACCATGGCTACCACCGCA	56.27	61
SCoT35	CATGGCTACCACCGGCCC	57.9	72
SCoT36	GCAACAATGGCTACCACC	51.53	56

### Selection, Cloning, and Sequencing of Species-Specific SCoT Fragments

Any SCoT band present in a particular species and absent in all the other species was considered a species-specific marker. To verify the reproducibility of the results, SCoT-PCR amplifications were performed at least twice, and only repeatable amplicons were selected. A unique band specific to *D. officinale* was excised and purified from an agarose gel using a SanPrep Column DNA Gel Extraction Kit (Shanghai Sangon Biological Engineering Technology and Service Co., Ltd., Shanghai, China). The purified product was ligated into a pMD^TM^ 19-T vector (Takara Biomedical Technology Co., Ltd., Beijing, China) according to the manufacturer’s protocol and transformed into the ultracompetent *Escherichia coli* strain *Trans5*α using the heat shock method. The recombinant plasmids were isolated by red/white clone screening and sequenced bidirectionally using M13 universal primers at Shanghai Sunny Biotechnology Co., Ltd. (Shanghai, China).

### Designing and Validating SCAR Primers

The obtained sequence was edited using the online tool VecScreen^1^ to remove vector sequences. The obtained sequence was identified by performing a BLASTN-based search^2^ of the nucleotide databases, and it was deposited in GenBank (GenBank accession number: MN746373). Forward and reverse oligonucleotide SCAR primers were designed using Primer Premier 5 (Lalitha, 2000) based on the obtained sequence information. The SCAR primer pair SHF/SHR was developed using the sequenced SCoT fragment (Table 4). The primer lengths ranged from 18 to 24 bp, and the optimum annealing temperature (*T*_*m*_) value was adjusted to 65°C, with a range of 60 to 68°C. SCAR amplification was performed in 20 μL of reaction mixture containing 2 μL 1 × PCR buffer [100 mM (NH_4_)_2_SO_4_, 100 mM KCl, 1% Triton X-100, pH 8.8], 2 μL Mg^2+^ (25 mM), 0.8 μL dNTPs (10 mM), 1 μL of forward primer (10 μM), 1 μL of reverse primer (10 μM), 0.5 μL *Taq* DNA polymerase (2 U/μL) (TaKaRa Bio.), and 50 ng genomic DNA template. PCR analysis was performed in a thermal cycler (MJ Research PTC-100) using the following parameters: 5 min at 94°C; 35 cycles of 1 min at 94°C, 1 min at 65°C and 2 min at 72°C; and a final extension at 72°C for 10 min. The PCR products were run on 1.5% agarose gels and detected by GelStain (TransGen Biotech) staining. The specific amplified fragment was designated a species-specific SCAR marker.

**TABLE 4:** Characteristics of developed species-specific SCAR primer pair derived from cloned SCoT13-derived amplicon of *Dendrobium officinale*.

SCAR primer	SCAR primer sequence (5’–3’)	Length	Tm (°C)	GC content (%)	Working annealing temperature (°C)	Amplicon length (bp)
SHF	GGGGTCACTCTGGCTACG	18	61.2	66.67	65	197
SHR	TACGACATGGCGACCATC	18	60.3	55.56		

We also examined the sensitivity of the *D. officinale* genome DNA template concentration for SHF/SHR, and seven DNA template concentrations, specifically 0, 5, 10, 15, 20, 30, and 50 ng/μL, were set. To further verify the practicality of this marker, four *Dendrobium* samples of the *D. chrysotoxum*, *D. nobile*, *D. primulinum*, and *D. aduncum* species were utilized to simulate different adulterations of *D. officinale* (Table 5).

**TABLE 5:** Simulated adulteration samples used for SCAR marker SHF/SHR application.

Num.	Different incorporation ratio of *Dendrobium* species (%)					The total proportion of *Dendrobium* species adulteration (%)
	*D. officinale*	*D. chrysotoxum*	*D. nobile*	*D. primulinum*	*D. aduncum*	
1	100					0
2	50	50				50
3	33.3	33.3	33.3			67
4	25	25	25	25		75
5	20	20	20	20	20	80
6	0	25	25	25	25	100

## Results

### Species-Specific SCoT Primer and Specific Loci Identification

**FIGURE 1:**
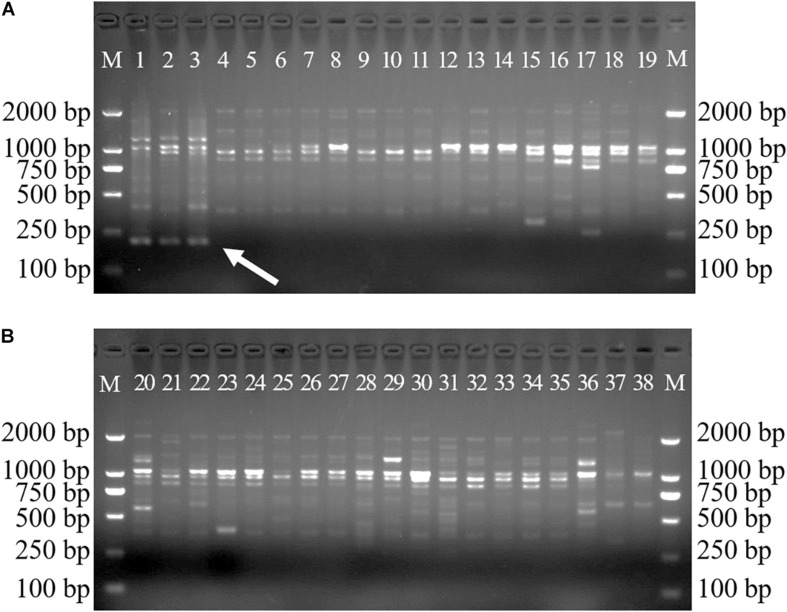
SCoT13 profiles of different Dendrobium species. Two 1.5% agarose gels were used for 38 Dendrobium species electrophoresis analysis **(A,B)**. Lane 1∼38: Details of 38 Dendrobium species are provided in Table 1. Lane M: Trans2K DNA Marker with lengths (bp); Arrowheads represent specific amplified bands in all the *D. officinale* individuals.

A total of 38 samples from 36 *Dendrobium* species, including most of the *Dendrobium* species that are easily confused with *D. officinale*, were chosen to develop species-specific SCAR markers for *D. officinale*. After screening, 22 SCoT primers with clear and repeatable polymorphisms were selected, and they yielded 337 loci, of which 324 loci were polymorphic and represented 96% of the polymorphisms. Among the 324 polymorphic loci, only primer SCoT 13 yielded a sharp and consistent DNA band of 215 bp that was unique to *D. officinale* samples (Figure 1), which could provide a molecular tool for the identification of *D. officinale*.

### DNA Sequence of the Species-Specific Fragment and Development of SCAR Marker

**FIGURE 2:**
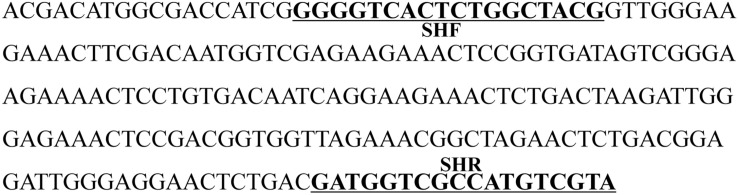
Nucleotide sequence of the SCoT marker specific to *D. officinale*. The sequence was named SCoT13-215, and has been deposited in GenBank (Accession number: MN746373), the underlined bold sequences represent the forward primer (SHF) and reverse primer (SHR).

The species-specific band obtained in all three *D. officinale* samples, named SCoT13-215, was cloned, sequenced, and deposited in GenBank (GenBank accession number: MN746373). The nucleotide sequence was 48.84% A + T and 51.16% G + C, as shown in Figure 2. The BLAST results revealed that the sequence had no homology with other sequences in GenBank, and no repeats were observed within the sequence. A SCAR primer pair, named SHF/SHR, was developed using Primer 3 based on the sequence SCoT13-215 (Table 4 and Figure 2).

### SCAR-PCR Amplification of the Designed Species-Specific SCAR Primer

**FIGURE 3:**
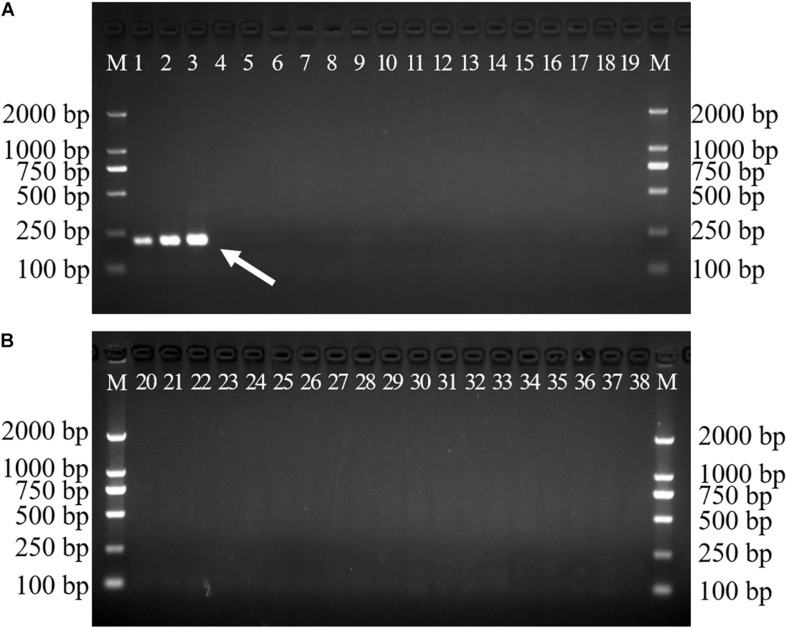
Amplification of the developed SCAR marker SHF/SHR in 38 different *Dendrobium* species. Two 1.5% agarose gels were used for 38 Dendrobium species electrophoresis analysis **(A,B)**. Lane 1∼38: Details of 38 Dendrobium species are provided in Table 1. Lane M: Trans2K DNA Marker with lengths (bp); Arrowheads represent specific amplified bands in all the *D. officinale* individuals.

**FIGURE 4:**
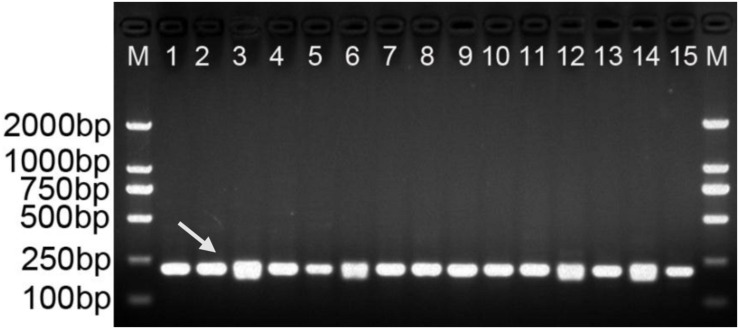
Amplification profiles of the primer pair SHF/SHR in 15 *D. officinale* individuals. Lane 1∼15: Details of 15 D. officinale individuals are provided in Table 2. Lane M: Trans2K DNA Marker with lengths (bp); Arrowheads represent specific amplified bands in all the *D. officinale* individuals.

Gradient PCR was performed to determine the best annealing temperature of the SCAR primers within the range of 60 to 68°C, and the optimal annealing temperature was determined to be 65°C. The newly developed SCAR primer pair SHF/SHR was validated by amplifying the sample DNAs listed in Table 1 at the optimal annealing temperature of 65°C. All the samples belonging to *D. officinale* were amplified and produced a sharp unique band of 197 bp, but no amplicons were produced for the other *Dendrobium* species (Figure 3). To further verify the stability and specificity of this SCAR marker, a PCR analysis of 15 different *D. officinale* samples (Table 2) using the primer pair SHF/SHR was performed, and all samples of *D. officinale* samples produced the specific amplicon at 197 bp (Figure 4). Thus, the SCAR primer pair (SHF/SHR) developed in this study was suggested to be a species-specific marker of *D. officinale*.

### Practicality of the Species-Specific SCAR Marker SHF/SHR

**FIGURE 5:**
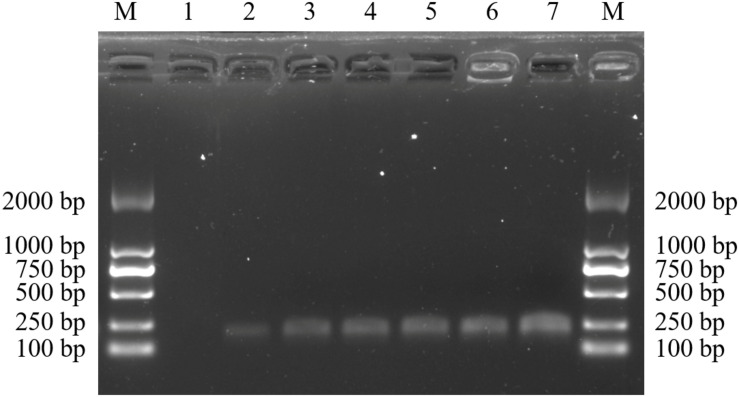
Amplification profiles of the primer pair SHF/SHR in different *D. officinale* genome DNA concentrations. Lane M: Trans2K DNA Marker with lengths (bp); Lane 1–7: 0, 5, 10, 15, 20, 30, and 50 ng/μL of *D. officinale* DNA template concentration.

**FIGURE 6:**
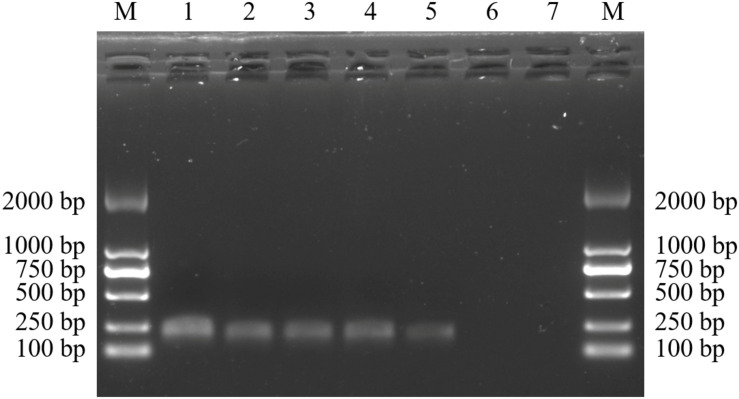
Amplification profiles of the primer pair SHF/SHR in different genome DNA composition of *Dendrobium* species. Lane M: Trans2K DNA Marker with lengths (bp); Lane 1–7: different proportions of *Dendrobium* species adulteration provided in Table 5.

After diluting the *D. officinale* DNA template concentration to 5, 10, 15, 20, 30, and 50 ng/μL, our species-specific SCAR marker SHF/SHR produced a distinct, 197 bp band (Figure 5). This finding means that DNA concentrations as low as 5 ng are sufficient for *D. officinale* detection in practical applications. In addition, only the simulated samples containing *D. officinale* could detect the 197 bp band of the SCAR marker SHF/SHR, whereas this band was not detected in the adulterated samples of *D. chrysotoxum*, *D. nobile*, *D. primulinum*, and *D. aduncum* (Figure 6). Thus, this marker could provide a scientific tool for determining whether *D. officinale* is present in Chinese medicinal materials for clinical use.

## Discussion

Species identification is necessary and important for product quality control, especially when the wrong species is deliberately provided. The stems of *D. officinale* are a rare traditional Chinese medicine, but in recent years, problems in the *D. officinale* market have become serious. Because of the numerous associated business entities, disordered competition, and similarity between the genuine *D. officinale* and its adulterants, it is highly common for other inferior *Dendrobium* species to enter the market as legitimate *D. officinale*, which has seriously affected the quality of *D. officinale* products on the market. Therefore, the authentication and traceability of *D. officinale* products are very important because of the increasing demand and the need to maintain the consumer quality of the products.

At present, many different DNA marker types, such as RAPD, ISSR, AFLP, and SSR, are available for the identification of medicinal plants, and they are also used in other fields, including taxonomy, physiology, and embryology (Teixeira da Silva et al., 2016). The identification of DNA molecular markers is particularly important when morphological identification is difficult, especially when samples are incomplete or damaged or the plants have become desiccated (Feng et al., 2018). Compared with the above-mentioned types of molecular markers, SCAR markers exhibit the advantages of high reliability and high detection sensitivity. In general, SCAR markers can be transformed from traditional DNA molecular markers, such as RAPDs (Zhang et al., 2015), AFLPs (Julio et al., 2006), SRAPs (Ji et al., 2014), and ISSRs (Albani et al., 2004). To increase the specificity of the amplification products, the sequence size of the PCR products amplified by SCAR primers is generally shorter than the original specific SCoT fragment. In recent years, an increasing number of SCAR markers have been designed for the identification of various medicinal plants, such as *Akebiae Caulis* (Moon et al., 2015), *Ganoderma lucidum* (Khan et al., 2016), *Saraca asoca* (Vinay, 2016), *Physalis* species (Feng et al., 2018), and *Panax ginseng* (Jiang et al., 2018).

Start codon-targeted markers are very popular because of their outstanding stability and polymorphic nature. SCoT-based SCAR markers have been used in the identification of many plant species or varieties, such as areca nut cultivars (Rajesh et al., 2016), *Taxus media* (Hao et al., 2018), and *Physalis* species (Feng et al., 2018). In our earlier study, we evaluated the genetic relationships of 36 *Dendrobium* species based on SCoT markers, and the findings indicated that a high degree of genetic diversity exists among Chinese *Dendrobium* species (Feng et al., 2015a). In this study, a specific 215 bp DNA sequence (SCoT13-215) of *D. officinale* obtained by the SCoT13 primer could be used for the identification of *D. officinale* by being transforming into a SCAR marker, and the specific band size amplified by the corresponding SCAR primer pair SHF/SHR was 197 bp, which was 18 bp shorter than the original specific SCoT sequence fragment.

The genus *Dendrobium* includes many species with different medicinal values, and *D. officinale* has the greatest value and accounts for a significant share in the global Chinese herbal medicine industry. The high value of *D. officinale* has resulted in the substitution of other *Dendrobium* species in place of *D. officinale*. To ensure the safe clinical use of *D. officinale* products, authentic raw materials are necessary. Thus, it is very important to develop a rapid and accurate method to identify *D. officinale* from its adulterants. The SCAR marker SHF/SHR developed in this study was able to amplify specific DNA fragments of a certain length in *D. officinale* samples, while no DNA bands were amplified in non-target *Dendrobium* species, which suggests that the SCAR marker can be used to distinguish *D. officinale* from other similar *Dendrobium* species. In addition, a minimum DNA template concentration of only 5 ng is sufficient for detection, which indicates that this method is highly sensitive.

## Conclusion

A specific DNA sequence, SCoT13-215, was obtained for *D. officinalis*, and the SCAR primer pair SHF/SHR was developed for species identification of *D. officinalis* in this study. The SCAR marker was proven to be a potential tool for the rapid, effective, and reliable determination of *D. officinale*, which may be highly useful for ensuring the quality of medicinal preparations and protecting this valuable medicinal species’ germplasm.

## Data Availability Statement

The datasets presented in this study can be found in online repositories. The names of the repository/repositories and accession number(s) can be found in the article/supplementary material.

## Author Contributions

JL and SF conceived and designed the experiments, participated in the analysis, and drafted the manuscript. KZ, YC, and YG performed the experiments. JJ performed the statistical analysis. WC and HW collected the plant samples. All authors read and approved the final manuscript.

## Conflict of Interest

The authors declare that the research was conducted in the absence of any commercial or financial relationships that could be construed as a potential conflict of interest.

## Footnotes

1 https://www.ncbi.nlm.nih.gov/tools/vecscreen/

2 https://blast.ncbi.nlm.nih.gov/Blast.cgi
